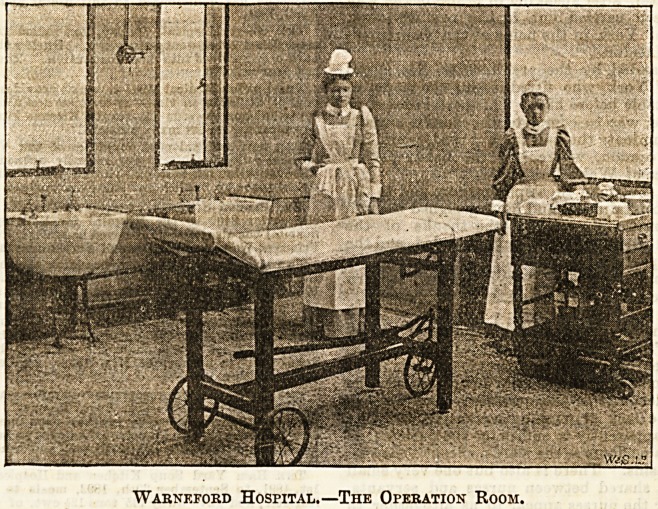# The Warneford Hospital, Leamington

**Published:** 1893-01-14

**Authors:** 


					256 THE HOSPITAL. Jan. 14, 1893.
HOSPITAL CONSTRUCTION.
THE WARNEFORD HOSPITAL, LEAMINGTON.
This very excellent institution appears to have grown out
of a charity established for the purpose of giving free baths
of the well-known Spa waters to poor patients. " The
Leamington Spa Charity," as it was called, was founded by
Benjamin Satchwell, the poet, in 1806, and was in 1826
merged with a dispensary and infirmary then carried on in
Regent Street. As the population of Leamington grew with
the increasing fame of its saline waters, tbe house in Regent
Street became inadequate for the growing work cf the
charity, and in 1831 a public meeting was held in order to
establish an infirmary on a wider and firmer basis. About
this time the Rev. Dr. Warneford, rector of Bourton-on-the-
Hill, was staying at Leamington, and interested himself in
the scheme to such good purpose that within a short time he,
on his own behalf and that of his sister,'added to the funds
of the hospital a total sum of ?2,500. And not alone by gifts
of money did Dr. Warneford show his interest in the work,
but, being elected on the committee, he gave liberally of his
time until age and infirmity precluded his further attend-
ance. On his death it was found that he had bequeathed
?10,000 upon trust for the benefit of the hospital under
certain conditions.
The site in the Radford Road upon which the hospital
stands was purchased in 1832, and the erection of the new
buildings proceeded with. The new hospital which was
opened in 1834 comprised what is now the central block of the
old building, and contained six wards, with accommodation
for twenty-four beds, four of which were in accordance with
a stipulation made by Dr. "Warneford, to be appropriated to
patients from the Radcliffe Infirmary, Oxford, and four for
bathing patients. In 1835 the Leamington Charity Bathing
Irstitution was amalgamated with the hospital. It was not,
however, until 1856 that funds were forthcoming for furnish-
ing and opening the whole of the wards, and in this year the
east wing, containing the outpatients' department and
liundry, were built.
In 1862 the buildiDg which is now the home for nurses was
erected as a sanatorium. In 1867, through the help of the
Warneford Trustees, the chapel was built, and in the follow-
ing year the west wing was erected. The hospital now with
these various additions possessed eighty-six beds in the main
bnilding and twenty in the sanatorium.
In 1877 the committee wisely decided to remove the out-
patient department from within the walls of the'hospital,
and accordingly built the one storey detached wing in which
this part of the work is at present carried on. In 1880 the
women's ward, with the children's ward above, was built,
and a new laundry and covered way to the nurse3' home
(late sanatorium) were built.
A new cottage hospital for the treatment of infectious
tevera was built in 1884 by the Rev. J. A. Beaumont, as a
memorial to hia son, after whom it is called the "Herbert)
Beaumont Cottage Hospital."
In 1890 the committee, having purchased additional land
to the west of the hospital, determined to build additional
wards and an operation-room, and at the same time to
re-organize the internal arrangements of the old house and
to enlarge the nurses' home.
The plans we publish show the ground floor of the hospital
with the new wing and operation-room. and the outline of
the proposed new wing to the south of the operation-room,
and the ground plan of the nurses' home.
The new ward wing is two storeys in height, and contains
on each floor a ward for fourteen beds, a separation ward
for one bed, nurses' duty-room, store-rooms for linen,
patients' clothes and food, and the usual sanitary offices.
HOSPITAL CONSTRUCTION ing year the west wing waa erected. The hospital now with
' ' these various additions possessed eighty-six beds in the main
THE WARNEFORD HOSPITAL, LEAMINGTON. building and twenty in the sanatorium.
' This very excellent institution appears to have grown out In 1877 the committee wisely decided to remove the out-
of a charity established for the purpose of giving free baths patient department from within the walls of the'hospital,
of the well-known Spa waters to poor patients. "The and accordingly built the one storey detached wing in which
Leamington Spa Charity," as it was called, was founded by this part of the work is at present carried on. In 1880 the
Benjamin Satchwell, the poet, in 1806, and was in 1826 women's ward, with the children's ward above, was built,
merged with a dispensary and infirmary then carried on in and a new laundry and covered way to the nurses' home
Regent Street. As the population of Leamington grew with (late sanatorium) were built.
the increasing fame of its saline waters, the house in Regent A new cottage hospital for the treatment of infectious
Street became inadequate for the growing work cf the
charity, and in 1831 a public meeting was held in order to .... r> n r
establish an infirmary on a wider and firmer basis. About
this time the Rev. Dr. Warneford, reotor of Bourton-on-the- tmtr
Hill, was staying at Leamington, and interested himself in
the scheme to such good purpose that within a short time he,
on his own behalf and that of his sister,'added to the funds
of the hospital a total sum of ?2,500. And not alone by gifts
of money did Dr. Warneford show his interest in the work,
but, being elected on the committee, he gave liberally of his
time until age and infirmity precluded his further attend-
ance. On his death it was found that he had bequeathed
?10,000 upon trust for the benefit of the hospital under
certain conditions.
The Bite in the Radford Road upon which the hospital
crcuho noon plan
The Warneford Hospital, Leamington.
Btands was purchased in 1832, and the erection of the new fevers was built in 1884 by the Rev. J. A. Beaumont, as a
buildings proceeded with. The new hospital which was memorial to his son, after whom it is called the "Herbert
opened in 1834 comprised what is now the central block of the Beaumont Cottage Hospital."
old building, and contained six wards, with accommodation In 1890 the committee, having purchased additional land
for twenty-four beds, four of which were in accordance with to the west of the hospital, determined to build additional
a stipulation made by Dr. Warneford, to be appropriated to wards and an operation-room, and at the same time to
patients from the Radcliffe Infirmary, Oxford, and four for re-organize the internal arrangements of the old house and
bathing patients. In 1835 the Leamington Charity Bathing to enlarge the nurses' home.
Irstitution was amalgamated with the hospital. It was not, The plans we publish show the ground floor of the hospital
however, until 1856 that funds were forthcoming for furnish- with the new wing and operation-room, and the outline of
ing and opening the whole of the wards, and in this year the the proposed new wing to the south of the operation-room,
east wing, containing the outpatients' department and and the ground plan of the nurses' home.
laundry, were built. The new ward wing is two storeys in height, and contains
In 1862 the building which is now the home for nurses was on each floor a ward for fourteen beds, a separation ward
erected as a sanatorium. In 1867, through the help of the for one bed, nurses' duty-room, store-rooms for linen,
Warneford Trustees, the chapel was built, and in the follow- patients' clothes and food, and the usual sanitary offices.
Jan. 14. 1893. THE HOSPITAL. 257
The wards have wax-polished oak floors, and the walls are
painted and varnished. Each ward is warmed by three
Boyd's hygiastic grates, two of them beiDg placed in the
centre of the ward and having descending flues. The sani-
tary offices are lined throughout with glazed bricks. The
sink-rooms are fitted with McETardy slop sinks,* and the
w.c.'s with Hellyer's pedestal hygienic apparatus.
Underneath the ground floor of the ward block is a base-
ment storey, the part of which that is under the main ward
being open at the Bides, the remainder being fitted up as
store-rooms, &c. As the groundffloor is raised^some feet
above the ground level, this arrangement, besides affording
a sheltered ambulatory for patients, allows a free sweep of
air under the ward floor from aide to side.
A one storey corridor, which will in the future extend
to the new south wing, gives access to the operation
room. This room has been constructed with a'view to its
being aB far as possible aseptic. The floor is of marble
" mischiati " mosaic, the walls are lined with glazed tiles,
the ceiling is painted, the window frames are of iron, and
the door of oak. It is fitted with two sinks"and a wash-basin
of white porcelain, all of which have tops of thick plate glass.
it is warmed by
a specially made
copper hot water
coil, and lit by a
pendant of three
Sugg's " Cro-
martie " gas bur-
ners, the pro-
ducts of combus-
tion from which
are conducted
into the open
air.
In the sur-
geons' room ad-
joining is a spe-
cially designed
instrument case
made throughout
of wainscot.
The only struc-
tural alteration
to the old house
consisted of the
erection of the
new sanitary
wing to the women's accident ward and the children s
Ward above.
In expending money upon alterations the committee laid
great stress upon the necessity for improving the accommoda-
tion for their nursing staff. One great need was a suitable
sitting room for nurses when off duty. This was made by
appropriating part of the men's ward on the ground floor of
the east wing. This room looks due south, and makes a very
pleasant and suitable room for the purpose, and the old
sanitary offices adjoining being cleared out the space forms
an excellent book-room. The remainder of this ward has
been converted into a much needed linen-room, with a small
office for the Matron adjoining.
The nurses' home has been enlarged by the addition of
twelve bed-rooms and two bath-rooms.
In addition to these alterations the whole of the hot-water
services, both for warming and for baths, &c., has been re-
organised, and two new powerful boilers laid down ; and the
drains to the old house, which were examined by Dr. Geo.
Wilaon in conjunction with the architect, were relaid.
The whole of the works were carried out^from the designs
of Mr. Keith D. Young.
. An illustration and description of this apparatus appeared in our
issue of May 145h, 1892.5^
Warneford Hospital.?The Operation Room.

				

## Figures and Tables

**Figure f1:**
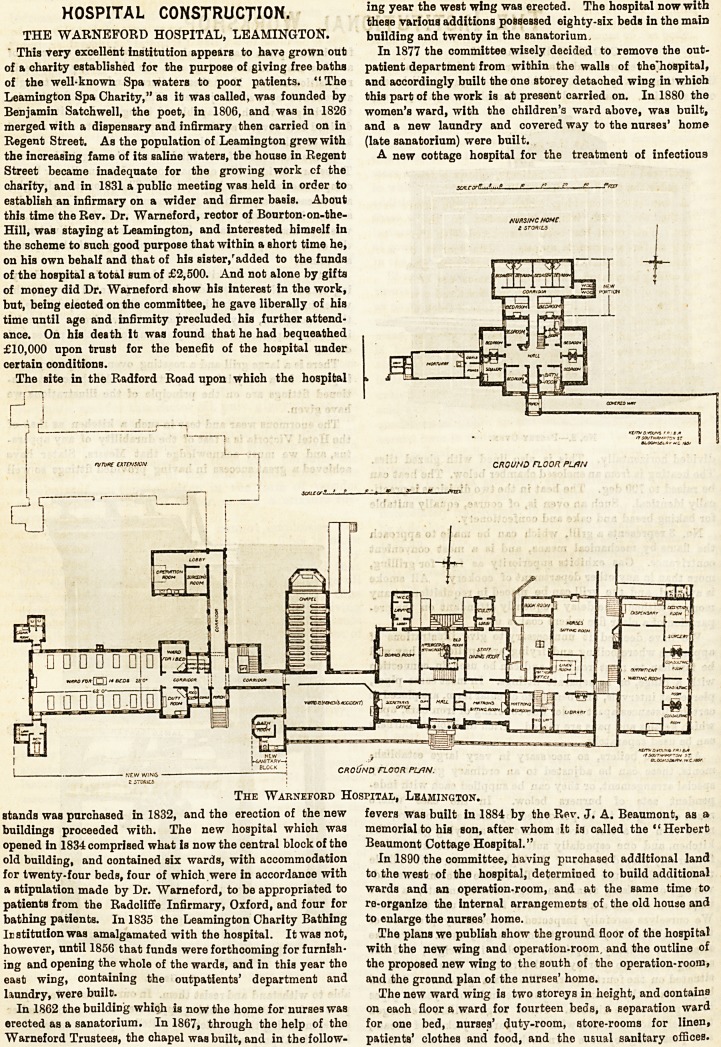


**Figure f2:**